# Two-stage revision and systemic antifungal therapy of *Candida glabrata* primary prosthetic hip infection successfully treated: a case report

**DOI:** 10.1186/s13256-019-2095-7

**Published:** 2019-05-21

**Authors:** Maria Bruna Pasticci, Chiara Papalini, Andrea Leli, Gastone Bruno

**Affiliations:** 1 Infectious Disease Clinic, Medicine Department, University of Perugia, Perugia, Italy; 2 Orthopedic Unit, Branca Hospital, Azienda Sanitaria Umbria 1, Perugia, Italy

**Keywords:** *Candida glabrata*, Prosthetic hip infection, Prosthetic joint infection, Echinocandin, Therapy, Surgery

## Abstract

**Background:**

Overall, fungi are estimated to cause approximately 1% of prosthetic joint infections, *Candida glabrata* account for less than 10% of these cases. No well-defined treatment strategy is available.

**Case presentation:**

A 71-year-old Caucasian man with non-insulin-dependent diabetes was admitted for hip prosthesis revision. For the past 17 years he suffered from recurrent infection of a perianal fistula, the last episode being 1 week before admission, and was prescribed amoxicillin/clavulanate 1 g twice a day. At surgery, the synovial fluid tested positive for infection with the Synovasure® Alpha Defensin Test, and the orthopedic surgeon reported intraoperative evidence of infection. While the synovial fluid failed to grow microorganisms, seven different samples including periprosthetic tissue and the prosthesis grew *Candida glabrata*. Imipenem 2 g and teicoplanin 600 mg daily were administered during surgery. Also an antibiotic loaded spacer was positioned. A week later micafungin 100 mg a day was added, and after another week imipenem was replaced with ertapenem 1 g once a day. The combination of antibiotics and antifungal was administered for a total of 7 weeks, while he also underwent treatment of the perianal fistula. The reimplantation was performed after an 8-week antibiotic-free interval. Before reimplantation, his erythrocyte sedimentation rate and C-reactive protein level were normal. At reimplant surgery, several samples were collected for microbiology, before administering ertapenem 1 g, teicoplanin 600 mg and micafungin 100 mg once a day. This antimicrobial combination was continued for 15 days until the microbiologic investigations, including culture and molecular testing after sonication technique of the spacer, were reported negative for bacteria and fungi. In this patient, systemic antifungal and extensive debridement allowed for clinical and microbiologic cure.

**Conclusions:**

Although *Candida glabrata* prosthetic joint infection is a rare event, the incidence could increase in the future, and there is need for more definitive treatment protocols. Diagnosis depends on culture. Fungal etiology must always be included in the differential diagnosis of prosthetic joint infection.

## Background

Prosthetic joint infections (PJI) occur in approximately 1% of joint replacements; they are less frequent in hip than in knee or shoulder arthroplasty. Their burden is on the rise due to the increased need for joint replacements and revisions, increased length of time the prosthesis remains *in situ*, increasing number of patients with risk factors for infection, and improved methods of diagnosis [1–3].

Gram-positive bacteria are the most frequent microorganisms causing PJI. Gram-negative bacteria account for approximately 5–10% of the cases. In 2–10% of the patients no microorganisms are identified and in another 5–10% there is more than one microorganism. Overall, fungi, primarily *Candida* species, are estimated to cause approximately 1% of all cases [4–6]*.* In the review by Kuiper *et al.*, 88% of fungal PJI were caused by *Candida* species., with *C. albicans*, *C. parapsilosis*, and *C. glabrata* rated 53%, 27%, and 9.6%, respectively [6]. Similar rates are reported in the paper by Cobo *et al*., including 76 cases of *Candida* species PJI [4]. Among 77 patients diagnosed with PJI in our clinic, from 2014, *Candida* species were identified in two cases (2.6%). One of those is the patient reported here, and the other was a patient with early PJI caused by *Propionibacterium acnes*, complicated by *C. parapsilosis* superinfection (author, unpublished data).

While there are guidelines for bacterial PJI [1–3], no well-defined strategy is available for fungal PJI, and above all for *C. glabrata* PJI. In a review dated October 2018 a total of 15 cases of PJI caused by *C. glabrata* were examined [7]. Therefore, it is important for physicians to share experiences in treating these infections, to allow for earlier diagnosis, better treatment protocols, and more favorable outcomes.

We report a case of primary hip prosthesis infection caused by *C. glabrata*. In this patient, mycotic etiology was unexpected, and the diagnosis was delayed. Likewise, specific treatment was delayed until culture results were available. The patient was treated successfully with a two-stage exchange, extensive debridement, and a 7-week course of systemic antifungal treatment.

## Case presentation

A 71-year-old Caucasian man with non-insulin-dependent diabetes was admitted with a diagnosis of loosened right hip prosthesis. The prosthesis had been implanted 6 years earlier for degenerative joint disease.

He was a social drinker and did not smoke tobacco. He reported high blood pressure and dyslipidemia with high triglycerides and low high-density lipoprotein levels. The medical treatment consisted of: metformin 500 mg/day, ramipril 5 mg/day, and fenofibrate 145 mg/day.

**Fig. 1:**
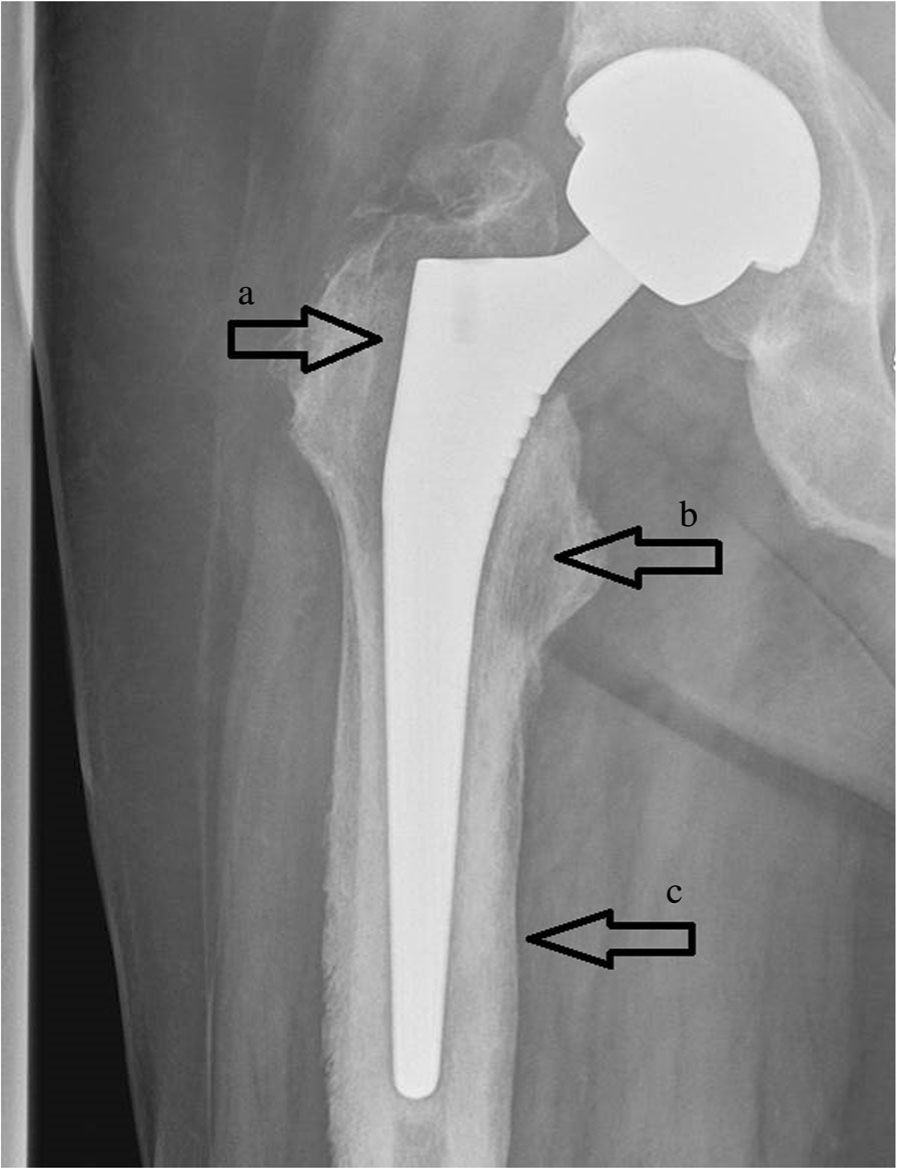
Hip radiography showing radiolucency lines at the proximal femoral/steam interface (arrows **a** and **b**), and periosteum reaction around the distal part of the stem (arrow **c**)

**Fig. 2:**
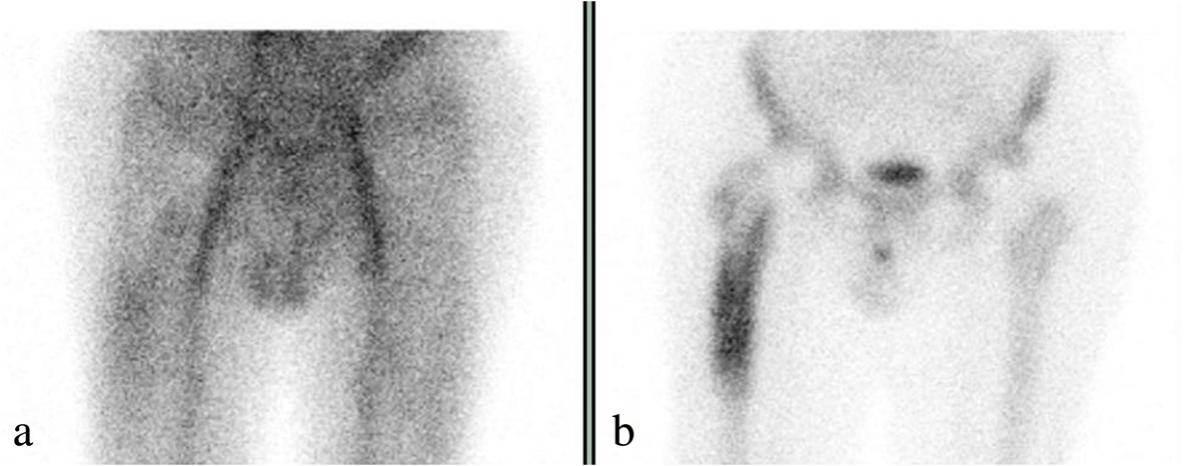
Triple phosphate bone scan. **a** Early phase – normal radionuclide distribution, and **b** delayed phase – fixation of the radionuclide at the proximal bone/stem interface

Four months earlier, he experienced a sudden onset of hip pain which became progressively worse. A hip radiograph showed radiolucency at the proximal femoral/stem interface (Fig. 1). A tri-phase bone scan evidenced normal distribution of the radionuclide at the early phase, and increased uptake at the delayed phase; the findings were judged non-indicative of infection (Fig. 2). On admission, he also reported to have suffered for the past 17 years from a perianal fistula, with recurrent flare ups of infection and multiple short courses of antibiotics. The last episode occurred 1 week before admission for which he was prescribed amoxicillin/clavulanic acid 1 g twice a day. A physical examination evidenced good general condition, normal temperature, blood pressure 130/70 mmHg, pulse rate 70, and O_2_ saturation 99%. No crepitations were present on auscultation in both lung bases; his abdomen was not distended, not tender, and bowel sounds were present. His liver and spleen were not enlarged. Cardiovascular and neurological systems examinations were normal. A draining perianal fistula was present. Also, pain of his right hip on leg motion and limited motion with lameness of his right leg were evidenced.

Laboratory examinations revealed: white blood cells (WBCs) 8240/mm^3^ with neutrophils 74%, erythrocyte sedimentation rate (ESR) 15 mm 1°hour, C-reactive protein (CRP) 1.32 mg/dL (normal range 0.1–0.75 mg/dL), alanine aminotransferase 16 IU/L (normal range 17–63), aspartate aminotransferase 18 IU/L (normal range 0–40), urea 23 mg/dL (normal range 12–71), and creatinine 0.36 mg/dL (normal range 0.9–1.3). The day after admission, he underwent prosthesis explant. The Synovasure® Alpha Defensin Test, CD diagnostics (Zimmer GmbH, Switzerland), was performed during surgery and it was indicative of infection [8, 9]. Purulent material was also evident around the acetabular cavity and the femoral diaphysis, and both prosthesis components were loosened and easily removed. A total of eight different samples, including the synovial fluid, the periprosthetic tissue, and the prosthesis, were sent for microbiological investigation. None was examined histologically. Thereafter, teicoplanin 600 mg twice a day was administered intravenously for the first day, followed by 600 mg a day administered intravenously and imipenem 500 mg four times a day administered intravenously, thus having empiric activity against Gram-positive bacteria as well as Gram-negative and anaerobic bacteria. The samples collected were transported without the use of specific transport media for anaerobic bacteria to the microbiology laboratory; however, cultures for aerobic and anaerobe bacteria and fungi on solid media were performed. When surgery was performed, blood cultures were not obtained, nor were cultures of the rectal swabs and the perianal fistula. A week later, from all samples examined, except the synovial fluid, *C. glabrata* grew on Sabouraud dextrose agar plates. The isolate was identified using the commercial VITEK® 2 card for yeast identification card (bioMérieux Diagnostic, Chemin de L’Orme, France). Antifungal susceptibility was determined evaluating the minimal inhibitory concentration (MIC), resulting in: fluconazole 4 mg/L, caspofungin ≤ 0.25 mg/L, micafungin ≤ 0.06 mg/L, and amphotericin B ≤ 0.25 mg/L (commercial VITEK® 2 system for susceptibility testing of yeast species, bioMérieux Diagnostic, Chemin de L’Orme, France). Micafungin 100 mg a day administered intravenously was added to the antibiotic regimen.

While on antibiotic and antifungal combination therapy, he underwent surgical treatment of the perianal fistula by positioning a cutting seton. When surgery was performed, rectal swab and perianal fistula cultures were not obtained. One week later, imipenem was replaced with ertapenem 1 g administered intravenously daily, to allow once a day out-patient treatment. Overall, the antimicrobial combination, including teicoplanin 600 mg administered intravenously, ertapenem 1 g administered intravenously, and micafungin 100 mg administered intravenously once a day, was administered for a total of 7 weeks. While on this therapy, he was monitored every 10 days for toxicity and efficacy, and teicoplanin blood levels ranged between 15 and 19 mg/L.

After 8 weeks of an antimicrobial-free interval, prosthesis reimplant was performed. Before reimplant, cardiac, abdominal, and eye fungal localizations were excluded, blood cultures resulted negative, and ESR and CRP were normal. At surgery, bone samples and the spacer were collected for microbiologic investigations, then teicoplanin 600 mg, ertapenem 1 g, and micafungin 100 mg once a day were administered intravenously. Two weeks later, the microbiologic reports failed to identify fungi or bacteria in all the samples cultured, including the spacer examined with and without the sonication technique [10], and with traditional as well as commercial real-time multiplex polymerase chain reaction assay LightCycler® testing (Roche Molecular Diagnostics, Mannheim, German) [11, 12]. Therefore, both antibiotic as well antifungal drugs were discontinued.

At follow-up, 24 months later, he was cured and free of pain. The prosthesis is functioning well, and ESR and CRP are within the normal range.

## Discussion

The authors report on a case of primary hip PJI caused by *C. glabrata*. In this patient as in the other published cases, the clinical manifestations were mild. Mycotic etiology was unexpected; the etiologic diagnosis was delayed, as well as specific treatment [4–7].

*C. glabrata* is less virulent than *C. albicans.* It is normally found in the gastrointestinal tract; it more commonly causes infections in aged or immunosuppressed people. *C. glabrata* fungemia is treated with fluconazole; it can be resistant to azole drugs when a patient has been exposed to these drugs [13].

*C. glabrata* was unexpected in this 71-year-old patient who was not immunocompromised and who did not have apparent traditional comorbidities associated with *Candida* infection like malignancy or immune deficiency. Other recognized risk factors for fungal infection and specifically infection with *Candida* species are diabetes, protracted antibacterial treatments, indwelling catheter, abdominal surgery, coexisting or previous bacterial PJI, and silent *Candida* bacteremia [4–6].

This patient had non-insulin-dependent diabetes that is unlikely to have predisposed him to *C. glabrata* infection. However, he had undergone repeated cycles of antibiotics that could have been the cause of *C. glabrata* gastrointestinal colonization, silent bacteremia, and, subsequently, infection of the prosthesis. The diagnosis of *C. glabrata* PJI was based on the results of Sabouraud dextrose agar plates culture. Colonies from Sabouraud agar plates were identified as *C. glabrata* using commercial cards for yeast identification. Also, antimicrobial susceptibility tests were obtained with commercial cards. The administration of an echinocandin was due to the MIC for fluconazole of the isolate [13].

*C. glabrata* was the only microorganism identified in seven out of eight intraoperative specimens, and it is likely that it was the only microorganism causing prosthesis infection in this patient. However, the decision was made to continue the antibiotic therapy in association with micafungin for the following reasons: (1) our patient’s history of recurrent perianal infected fistula; (2) he was prescribed amoxicillin/clavulanate until the day before the prosthesis was explanted and the samples for microbiologic investigations were obtained; (3) all the samples collected for microbiologic investigations were not transported to the laboratory with specific transport media for anaerobic bacteria; (4) the removed prosthesis was cultured without sonication technique; and (5) after the prosthesis was explanted he underwent perianal fistula surgery with the placement of a seton for over 4 weeks. The first of these points could represent a risk of involvement of Gram-negative and/or anaerobic gut bacteria as the etiology of PJI; furthermore, their recovery could be hampered given that he was on amoxicillin/clavulanate treatment until the day before the explant surgery, the collected samples were not transported with specific media for anaerobic bacteria to the microbiology laboratory, sonication technique cultures were not included in the microbiologic investigations [2, 3, 10], and, finally, the presence of a seton to treat the perianal fistula was considered a possible persistent source of bacterial infection for this high risk surgery.

According to the published literature, treatment of fungal PJI is difficult. The most widely adopted protocols include a two-staged treatment, extensive and radical debridement, systemic antifungal therapy, and a long interval without antimicrobial therapy before reimplantation [4–7, 14–16]. Some authors also suggested spacers loaded with antibiotics and/or antifungal drugs, but there is uncertainty with regard to the antifungal agent to be added to the cement [4–7, 14–16]. Moreover, neither the interval between explant and reimplant, nor whether an association of systemic antifungal therapy is more efficacious than a single antifungal agent has been clearly established [4–7, 14–16]. The optimal duration of antifungal treatment for the eradication of infection before and after reimplantation is also currently unknown [4–7, 14–21]. Some authors suggested a prolonged course of up to 1 year of therapy after reimplantation, especially in those patients at high risk for treatment failure or who could have a poor outcome [16–21]. In the review by Kuiper *et al.* on *Candida* PJI, duration of treatment ranged from none to more than 1 year, without reference to timing of reimplant surgery [6].

This patient underwent explant for a loosened prosthesis. The diagnosis of PJI was unexpected and the diagnosis of *C. glabrata* PJI was even more unexpected and also delayed. The radiography findings were suggestive of loosening of the prosthesis [22]. A positive scan with technetium can reflect increased bone activity; however, it lacks specificity for infection [23]. A preoperative joint aspirate was not done, yet it would have been of no help to plan a more appropriate treatment strategy. In fact, the synovial fluid aspirate was the only sample from which *C. glabrata* was not cultured. Synovial fluid culture has some limitation in the diagnosis of PJI [9], and this is true especially in cases caused by low virulence pathogens such as *C. glabrata*.

He was treated as follows: 7-week course of micafungin administered along with teicoplanin and ertapenem, followed by 8-week interval without antimicrobials; then, after the prosthesis was reimplanted, the antimicrobial therapy was administered for another 2 weeks, until the microbiologic results, including spacer sonication and SeptiFast testing on sonicated fluid, were reported negative [10–12]. Micafungin was well tolerated without side effects.

At follow-up of this patient, 24 months later, clinical and microbiologic cure was obtained with the administration of systemic micafungin for 7 weeks and extensive surgical debridement.

The Synovasure® test [8, 9] measures alpha defensin released by neutrophils in response to synovial fluid pathogens. It can predict PJI, yet it does not identify the etiologic agent. The test has been evaluated in patients with bacterial PJI with an overall sensitivity and specificity of 95%. There are no data regarding its performance in fungal PJI. In our patient, diagnosed with *C. glabrata* PJI, the alpha defensin test results indicated the presence of infection.

## Conclusion

In conclusion, *C. glabrata* PJI is a very rare event. However, the incidence could increase in the future, and there is need for more definitive treatment protocols. Diagnosis depends on culture. Fungal etiology must always be included in the differential diagnosis of PJI.
